# Efficacy of a home‐used high‐intensity focused ultrasound device on wrinkle reduction

**DOI:** 10.1111/srt.13266

**Published:** 2022-12-28

**Authors:** Mi Hee Kwack, Weon Ju Lee

**Affiliations:** ^1^ Department of Dermatology, School of Medicine, Kyungpook National University Kyungpook National University Hospital Daegu South Korea; ^2^ Department of Immunology School of Medicine Kyungpook National University Daegu South Korea; ^3^ BK21 FOUR KNU Convergence Educational Program of Biomedical Sciences for Creative Future Talents, School of Medicine Kyungpook National University Daegu South Korea

**Keywords:** collagen, elastin, home‐used high‐intensity focused ultrasound device, matrix metalloproteinase‐1, mice, tissue inhibitor of metalloproteinase‐1

## Abstract

**Background:**

High‐intensity focused ultrasound (HIFU) has been developed for the treatment of skin wrinkles on the face, neck, and body.

**Objectives:**

This study aimed to evaluate the effects of a home‐used HIFU device on wrinkles in mice based on the expression of fibrosis‐related genes and proteins.

**Methods:**

The backs of 20‐week‐old mice were treated with a home‐used HIFU using the following probes: 4 MHz, 1.5 mm focal depth. The treated mice were compared with young mice by histological examination, real‐time polymerase chain reaction (PCR), and immunohistochemistry. Histological examination was performed by trichrome staining. Real‐time PCR and immunohistochemistry were conducted to determine the expression of collagen types I and III, matrix metalloproteinase (MMP)‐1, and tissue inhibitor of metalloproteinase (TIMP)‐1.

**Results:**

Dermal thickness was increased after treatment with the home‐used HIFU device at 30 and 60 s per day for 1 week or 30 and 60 s per day for 2 weeks on trichrome. Gene and protein expression of collagen types I and III and elastin were increased after treatment with HIFU at all options of 30 and 60 s per day for 1 week or 30 and 60 s per day for 2 weeks. Gene and protein expressions of MMP‐1 and TIMP‐1 were decreased after treatment with HIFU device at 30 and 60 s per day for 1 week or 30 and 60 s per day for 2 weeks.

**Conclusion:**

The home‐used HIFU device can be an effective therapeutic modality for skin tightening.

## 1 INTRODUCTION

Skin wrinkles are part of the natural process of aging in humans, although sun exposure, pollutants, and smoking are major causes of premature skin wrinkles. Although it is inevitable to develop wrinkles, its appearance is highly disliked by the majority of people. This aversion for wrinkles has enabled the global antiaging market to expand significantly.

Therapeutic modalities for reducing skin wrinkles have been developed throughout the world.^1^ Botulinum toxin A is a popular anti‐wrinkle agent used to improve the appearance of aging skin.^2^ Frown lines and crow's feet are the cosmetic indications approved by the US Food and Drug Administration for botulinum toxin A. Dermal filler and thread lifting are also popular therapeutic tools for the treatment of skin wrinkles.^3^, ^4^ Treatment of skin wrinkles with laser devices is well known. The variety of laser devices that have been used include Er:YAG laser, fractional Er:YAG laser, CO_2_ laser, fractional CO_2_ laser, and picosecond laser.^5^, ^6^, ^7^ Only recently, radiofrequency therapy has been introduced in esthetic dermatology.^8^ Furthermore, mesotherapy, microneedling, and chemical peels were used as options for the treatment of aging skin.^9^, ^10^ Most of these therapeutic modalities are performed in general hospitals or private clinics.

The demand for safe and effective home‐used therapeutic devices for reducing skin wrinkles has been rising in recent years. Thus, we developed a new home‐used high‐intensity focused ultrasound (HIFU) device for skin wrinkle treatment. HIFU has been used in medicine to treat certain conditions, such as tumors. It has significant advantages over conservative treatment options using focused ultrasound energy.^11^ It treats patients using nonsurgical methods. Nevertheless, it is not used for brain disease because errors can occur for reasons such as sensory and gait disturbances until 2016. Currently, it overcame the problem and uses it to treat Parkinson's disease.^12^ Recently, commercially available HIFU devices are being developed for various therapeutic purposes. This device we made emits 4 Mhz HIFU and has a microneedle that can be adjusted according to the depth of the skin. The cartridge can be replaced, and the usage time and HIFU intensity can be adjusted. We tested the efficacy of our HIFU device against wrinkles by histological examination, real‐time polymerase chain reaction (PCR), and immunohistochemistry in a mouse model.

## 2 MATERIALS AND METHODS

### 2.1 Application of HIFU device on mouse skin

**FIGURE 1:**
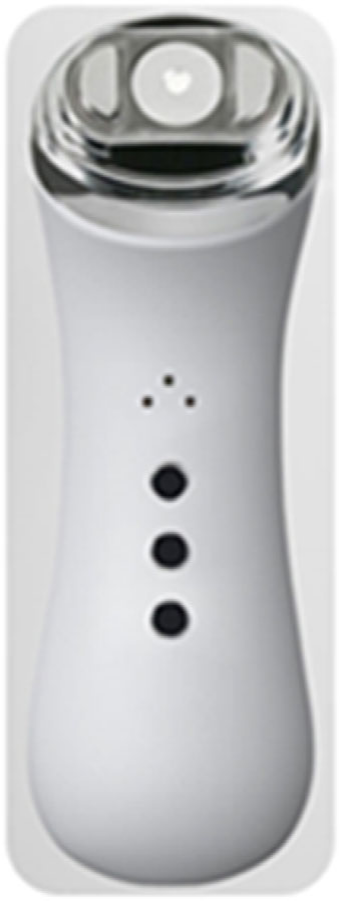
A novel high‐intensity focused ultrasound (HIFU) device

A novel HIFU device was provided by Research And Ubiquitous Inc. (Figure 1). This device allows the user to control the intensity, duration, and depth of the HIFU.

To study the effect of HIFU on mouse skin, nude female mice (*n* = 30) were purchased from Orient Bio Inc. (Sungnam, Republic of Korea) and were stabilized until 20 weeks in ventilated cages. Seven‐week‐old mice were used as a positive control group (*n* = 5). The experimental care and treatment were conducted in accordance with the ARRVE guidelines. The animal experiments were approved by the Institutional Animal Care and Use Committee of Kyungpook National University (Approval No. KNU 2021‐0184).

Old mice (20‐week old) were treated with HIFU 30 and 60 s per day for 1 week or 30 and 60 s per day for 2 weeks on the back skin. At 21 and 22 weeks, mice were sacrificed by CO_2_ inhalation after HIFU treatment. Pictures were immediately taken, and mouse skins were obtained.

### 2.2 Histological examination by trichrome staining

Excised mouse tissues were embedded in the optimum cutting temperature compound (Tissue‐Tek; Miles, Napierville, IL, USA). Tissues were cut to a thickness of 8 μm using a cryostat (Leica CM3050 S; Leica, Heidelberg, Germany). Slides were stained with trichrome, and immunohistochemistry was performed.

Trichrome staining kit (Scy Tek Laboratories, Logan, UT, USA) were used according to the manufacture's instructions to visualize collagenous connective tissue fibers in tissue sections. Briefly, the slides were fixed with preheated Bouin's Fluid for 60 min and then washed with water. After staining with hematoxylin mixture for 5 min, the slides were washed with water and placed in 0.5% acid alcohol solution for 5 s to adjust the pH. After staining with trichrome stain solution for 15 min and washing with water, the slides were rinsed in absolute alcohol for 10 s in 0.5% acetic acid solution. Blue indicates collagen, and red represents fibers.

Dermal thickness was measured in Trichrome‐stained images using a Zeiss micrometer (Carl Zeiss, Oberkochen, Germany) at 200× magnification.

### 2.3 Real‐time PCR

Total RNA was isolated from cutaneous dorsal mouse skin treated with HIFU using TRIzol reagent (Invitrogen, Waltham, MA, USA), and cDNA was synthesized using the cDNA synthesis kit ImProm‐II reverse transcriptase kit (Promega, Madison, WI, USA). Real‐time PCR was performed with 50 ng cDNA, 10 pM primers and SYBR Green I at 95°C for 10 min, 40 cycles at 95°C for 15 s, and 60°C for 60 s. The products of the PCR were quantified using the StepOnePlus Real‐Time PCR analysis software (Applied Biosystems). The primers sequences are showed in Table 1.

**TABLE 1:** Polymerase chain reaction (PCR) primers used in this study

Gene	Oligonucleotide primers	
Mouse *GAPDH*	AACTTTGGCATTGTGGAAGG	ACACATTGGGGGTAGGAACA
Mouse *collagen I*	GAGCGGAGAGTACTGGATCG	GTTCGGGCTGATGTACCAGT
Mouse *collagen III*	CACCTGCTCCTGTGCTTCCT	ACCTGGTTGTCCTGGAAGGC
Mouse *elastin*	GCTACTGCTTGGTGGAGAATG	CCCTTGGAGATGGAGACTGT
Mouse *MMP‐1*	GTTGGAGCAGGCAGGAAGG	TAGCAGCCCAGAGAAGCAAC
Mouse *TIMP‐1*	TCCCCAGAAATCAACGAGAC	CATTTCCCACAGCCTTGAAT

Abbreviations: MMP, matrix metalloproteinase, TIMP, tissue inhibitor of metalloproteinase.

### 2.4 Immunohistochemistry

Frozen section slides were fixed in 4% paraformaldehyde with 0.1% Triton X‐100 for 10 min. After 30 min of treatment in 3% H_2_O_2_ to remove nonspecific signals, blocking was performed in 5% donkey serum (Abcam, Cambridge, UK) for 1 h after washing. Sections were incubated with collagen type I (1:100 dilution; Invitrogen), collagen type III (1:100 dilution; Invitrogen), elastin (1:100 dilution; Bioss Antibodies, Woburn, MA, USA), matrix metalloproteinase (MMP‐1; 1:100 dilution; Invitrogen), and tissue inhibitor of metalloproteinase (TIMP‐1; 1:100 dilution; Bioss Antibodies) antibodies at 4°C overnight, washed three times with PBS, and incubated with horseradish peroxidase (HRP)‐conjugated donkey anti‐rabbit antibody for 1 h. After washing with PBS, AEC+ high sensitivity substrate chromogen (DAKO, Glostrup, Denmark) was used as color developer for HRP. Hematoxylin (DAKO) was used for counterstaining.

### 2.5 Statistical analysis

All statistical analyses were performed using the SPSS 22.0 software (IBM, Armonk, NY, USA). The results were expressed as the mean ± standard error of means. The results of multiple group analysis were analyzed using one‐way analysis of variance. Data were a representative of at least two independent experiments. Differences were considered to be statistically significant differences where *p* < 0.05.

## 3 RESULTS

### 3.1 Skin wrinkles were decreased after treatment with the HIFU device

**FIGURE 2:**
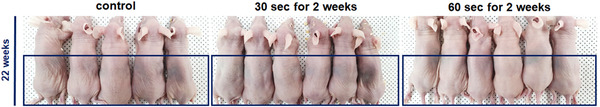
Skin wrinkles were decreased after treatment with the high‐intensity focused ultrasound (HIFU) device on mice

Immediately after scarifying mice, images of the back skin mice treated with HIFU device were taken and compared to the vehicle control at the same magnification and location (Nikon D7000, Japan). Mouse skin wrinkles were decreased after treatment with the HIFU device at both 30 and 60 s for 2 weeks compared with control (Figure 2).

### 3.2 Dermal thickness was increased after treatment with the HIFU device

**FIGURE 3:**
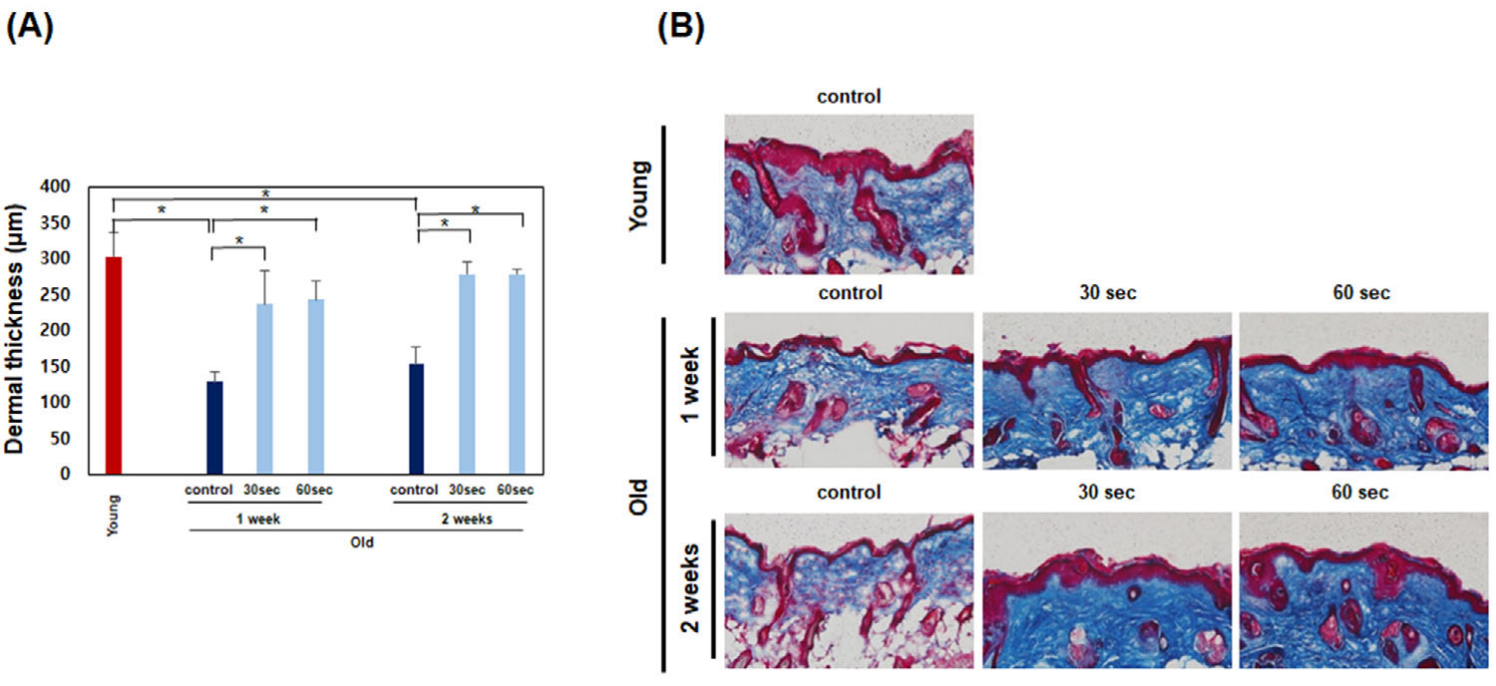
Measurement of dermal thickness after treatment with the high‐intensity focused ultrasound (HIFU) device (30 or 60 s per day for 1 week, 30 or 60 s per day for 2 weeks). (A) Dermal thickness was increased after all treatment options with the HIFU device. Data are the means ± SEM (*n* = 5) (*p < 0.05). (B) Histological examination by trichrome staining showed a denser and thicker dermis after all treatment options with the HIFU device

As the skin develops dermal thinning with age, wrinkles appearance tends to increase. As previously reported, the dermal thickness of old mice was thinner than that of young mice. Dermal thickness was increased after all treatment options with the HIFU device at of 30 and 60 s for 1 week or 30 and 60 s per day for 2 weeks in old mice (*p < 0.05) (Figure 3A). No significant difference was found between the 30 and 60 s per day treatments. In addition, no significant difference was found between the 1‐ and 2‐week treatment periods. Furthermore no significant differences were found between young mice and all treated mice.

Next, trichrome staining was used to analyze the changes in arrangement and structure of collagen. Histological examination by trichrome staining showed that the dermis was denser and thicker in all mice that underwent all treatment options (Figure 3B). The dermis of young mice and all treated mice were all similar on histological examination.

### 3.3 Collagen fibers and elastin were increased after treatment with the HIFU device

**FIGURE 4:**
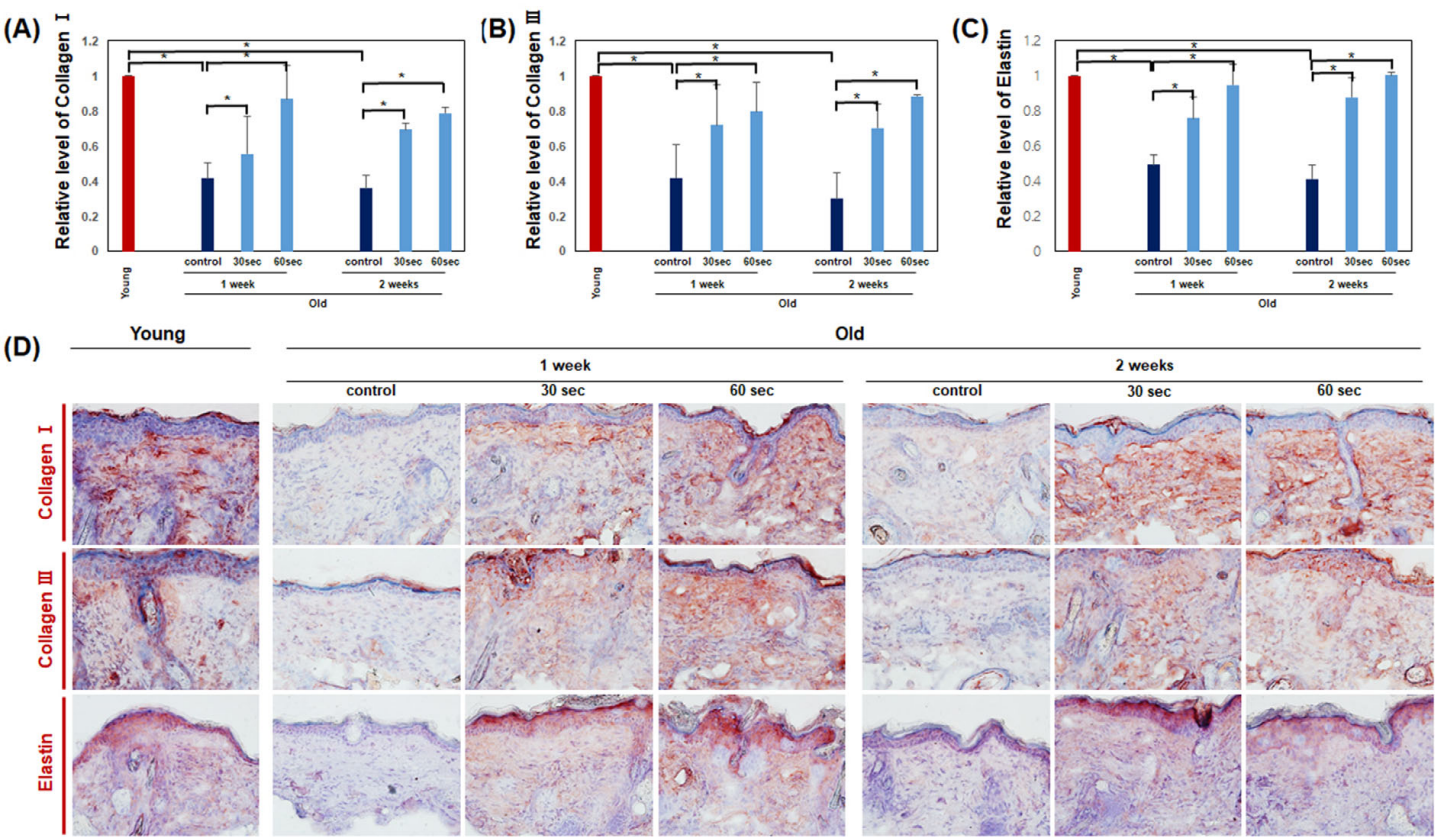
Expression of collagen fibers and elastin after treatment with the high‐intensity focused ultrasound (HIFU) device (30 or 60 s per day for 1 week, 30 or 60 s per day for 2 weeks). (A–C) Gene expressions of *collagen types* I and III and *elastin* were increased after all treatment options with HIFU device. The mRNA expression levels were measured by qPCR and normalized with *GAPDH*. Data are the means ± SEM (*n* = 5) (*p < 0.05). (D) Protein expressions of collagen types I and III and elastin were increased after all treatment options with HIFU device. Representative histologic section findings of mouse tissue immunostained with collagen type I (upper panels), collagen type III (mid panels) and elastin (lower panels) at 1 and 2 weeks after HIFU treatment

Gene expressions of *collagen types* I and III and *elastin* were increased after all treatment options with the HIFU device at 30 and 60 s per day for 1 week or 30 and 60 s per day for 2 weeks (*p < 0.05) (Figure 4A–C). No significant differences were found between the 30 and 60 s per day treatment. In addition, no significant difference was found between the 1‐ and 2‐week duration. Furthermore, no significant differences were found between young mice and all treated mice.

Immunohistochemical findings revealed that protein expressions of collagen types I and III and elastin were increased after all treatment options with an HIFU device at 30 and 60 s per day for 1 week or 30 and 60 s per day for 2 weeks compared with control mice (Figure 4D). Furthermore, protein expressions of collagen types I and III and elastin were similar between young mice and all treated mice.

### 3.4 MMP‐1 and TIMP‐1 were decreased after treatment with an HIFU device

**FIGURE 5:**
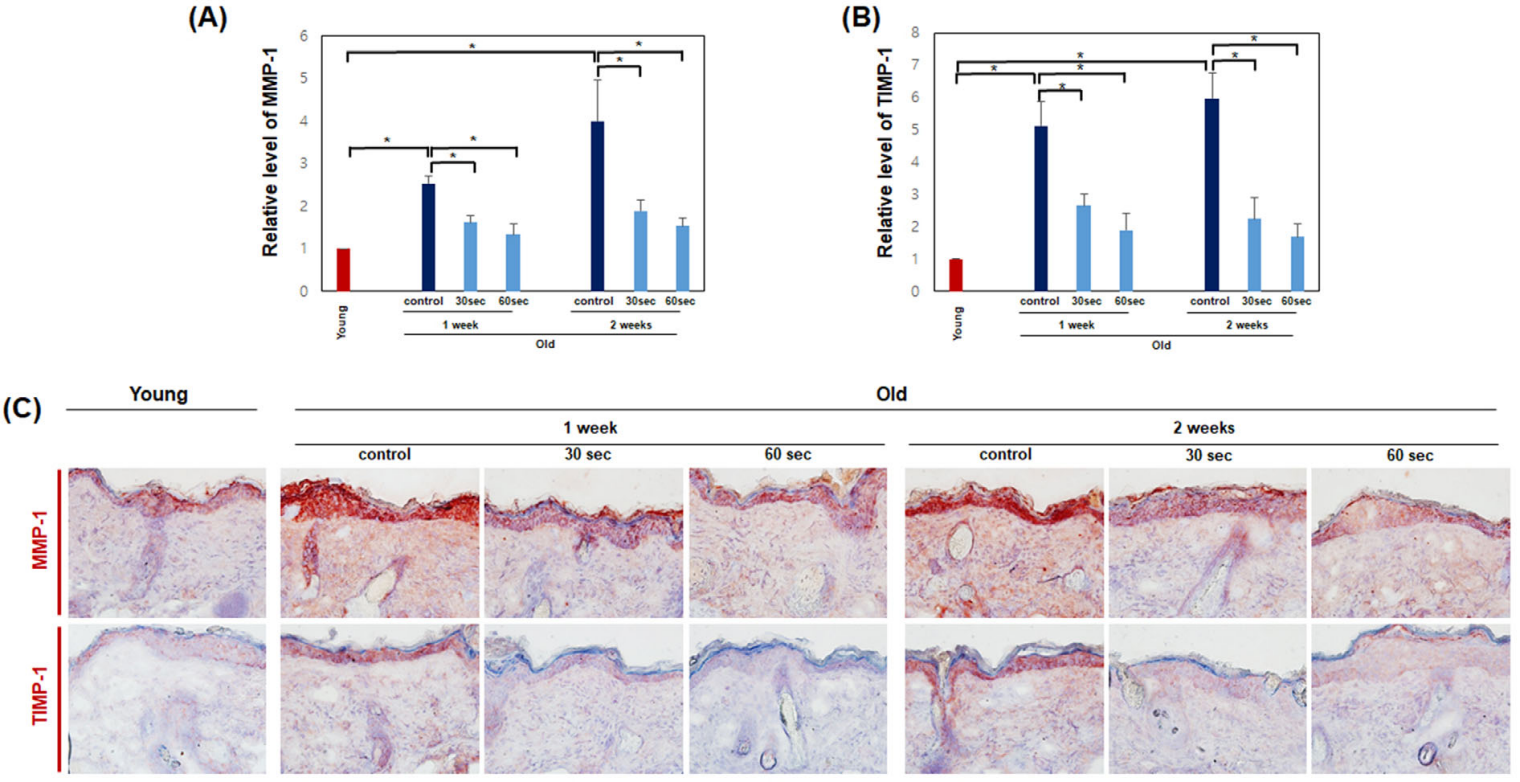
Expression of matrix metalloproteinase (MMP)‐1 and tissue inhibitor of metalloproteinase (TIMP)‐1 after treatment with the high‐intensity focused ultrasound (HIFU) device (30 or 60 s per day for 1 week, 30 or 60 s per day for 2 weeks). (A and B) Gene expressions of *MMP‐1* and *TIMP‐1* were decreased after all treatment options with HIFU device. The mRNA expression levels were measured by qPCR and normalized with *GAPDH*. Data are the means ± SEM (*n* = 5) (*p < 0.05). (C) Protein expressions of MMP‐1 and TIMP‐1 in the mouse skin were decreased after all treatment options with HIFU device. Representative histologic section findings of mouse tissue immunostained with MMP‐1 (upper panels) and TIMP‐1 (lower panels) at 1 and 2 weeks after HIFU treatment

Degradation of collagen and elastin in aged skin is related to increased MMP and TIMP expression. Gene expressions of *MMP‐1* and *TIMP‐1* were decreased after all treatment options with the device at 30 and 60 s per day for 1 week or 30 and 60 s per day for 2 weeks (*p < 0.05) (Figure 5A,B). No significant difference was found between the 30 and 60 s per day treatment, as well as differences were found between young mice and treated mice.

Immunohistochemical analysis showed that the protein expressions of MMP‐1 and TIMP‐1 in treated mice were decreased after all treatment options with an HIFU device at 30 and 60 s per day for 1 week or 30 and 60 s per day for 2 weeks than control mice (Figure 5C).

## 4 DISCUSSION

HIFU is one of the effective therapeutic modalities for skin wrinkles reduction. Many researchers have tried to develop an effective, simple, and practical HIFU for the reduction of skin wrinkles. Choi et al.^13^ reported that HIFU using transducers with a lower frequency and deep focal depth offers safe and effective treatment for dermal and subdermal tightening. In the study, all subjects were treated with HIFU to both cheeks, upper arms, lower abdomen, thighs, and calves using a variety of probes: 7 MHz, 1.5 mm focal depth; 2 MHz, 3.0 mm focal depth; 2 MHz, 4.5 mm focal depth; 2 MHz, 6.0 mm focal depth, and 2 MHz, 9.0 mm focal depth. They assessed results using the investigator and subject global aesthetic improvement scale, a cutometer, and a visual analog scale. Moreover, Jung et al.^14^ conducted a clinical trial comparing the efficacy and safety of two HIFU devices for facial skin tightening using qualitative and quantitative assessments of both clinicians and patients. They concluded that both HIFU devices can be used safely and effectively for facial skin tightening. Furthermore, Ko et al.^15^ reported that HIFU improves skin elasticity and clinical contouring of the face and body. Park et al.^16^ concluded that HIFU can be used to improved facial wrinkles and skin laxity in Asian skin, especially around the jawline, cheeks, and perioral areas.

In addition, researchers are trying to develop a device that combines the function of HIFU with other functions for better skin wrinkle improvement. Skin wrinkles due to multifactorial causes need a multifactorial approach for treatment.^17^ Combinations of neuromodulators, three‐ and two‐dimensional fillers, and energy‐based devices or surgical interventions can be used for treatment. HIFU can also be combined with dermal fillers for facial skin tightening.^18^ Nam et al.^19^ reported the synergistic effect of HIFU and low‐fluence Q‐switched Nd:YAG laser in the treatment of aging skin in the neck. In another study, the combination of HIFU and fractional CO_2_ laser resurfacing was safe and effective for lifting and tightening the face and neck.^20^ HIFU can also be applied with botulinum toxin and dermal filler to improve lines and wrinkles.^21^

Although numerous clinical trials on HIFU with or without combination therapy have been conducted, studies that evaluated the histological, immunohistochemical, and molecular effects of HIFU are limited. Casabona and Michalany^22^ performed a clinical and histological evaluation of neocollagenesis after treatment with microfocused ultrasound and filler. They showed the enhancement and quality of the new collagen and elastin fibers in histological findings. Interestingly, a report on changes in circulating immunosuppressive cytokine levels in cancer patients after treatment with HIFU was introduced.^23^

In this study, we demonstrated that the novel home‐used HIFU device we developed was safe and effective in the treatment of skin wrinkles. Skin wrinkles on mice were improved after treatment with the device. The improvement of skin wrinkles was proved with histological findings, real‐time PCR, and immunohistochemistry. Trichrome staining showed increased dermal thickness, whereas real‐time PCR and immunohistochemistry revealed an increase in the gene and protein expressions of collagen I and III and a decrease in the gene and protein expression of MMP‐1 and TIMP‐1. As previously reported, there are limitations in evaluating the anti‐wrinkle effect only with increased expression of collagen and elastin and decreased expression of MMP and TIMP, but these are currently used as wrinkle‐improving markers. Furthermore, it is necessary to gradually evaluate whether the HIFU we developed is effective for humans.

Based on these preliminary results in mouse, this HIFU device might prove useful in humans. Future studies are needed to evaluate whether these exploratory results can be extrapolated into humans.

## CONFLICTS OF INTEREST

The authors have no conflicts of interest to disclose.

## Data Availability

The data that support the findings of this study are available from the corresponding author upon reasonable request.
